# Trends and seasonal variation of hospitalization and mortality of interstitial lung disease in the United States from 2006 to 2016

**DOI:** 10.1186/s12931-020-01421-0

**Published:** 2020-06-16

**Authors:** An Thi Nhat Ho, Artem Shmelev, Edward Charbek

**Affiliations:** 1 Department of Pulmonary and Critical Care Medicine, Saint Louis University Hospital, Saint Louis, MO USA; 2 Department of surgery, Saint Agnes Hospital, Baltimore, MD USA

**Keywords:** Interstitial lung disease, Seasonal variation, Mortality, Hospitalization

## Abstract

**Background:**

In the recent years, the overall trends in hospital admission and mortality of interstitial lung disease (ILD) are unknown. In addition, there was some evidence that interstitial lung disease death rate highest in the winter but this finding was only available in one study. This study will investigate the trend and seasonal variations in hospital admission and mortality rates of ILD from 2006 to 2016.

**Method:**

From the Nationwide Inpatient Sample database, we collected all cases with the International Classification of Diseases (ICD)-9 or ICD-10 codes of ILD excluding identifiable external causes (drug, organic or inorganic dusts) from 2006 to 2016. Hospitalization rates of each year were calculated based on U.S Census population data. Monthly hospitalization and in-hospital mortality rates were analyzed by seasonal and trend decomposition. Subgroups of idiopathic interstitial fibrosis (IPF), acute respiratory failure (ARF), pneumonia were analyzed.

**Results:**

From 2006 to 2016, all-cause hospital admission rate of patients with interstitial lung disease (ILD) and IPF-only subgroup declined but their overall mortality remained unchanged (except IPF subgroup and acute respiratory failure subgroup). Acute respiratory failure related admission account for 23% of all causes and pneumonia 17.6%. Mortality of ILD in general and subgroup of ILD with ARF was highest in winter, up to 8.13% ± 0.60 and 26.3% ± 10.2% respectively. The seasonal variations of hospital admission and mortality of ILD in general was not changed when infectious pneumonia cases were ruled out. All cause admission rates were highest in months from January to April. Subgroup analysis also showed seasonal variations with highest hospitalization rates for all subgroups (IPF, ARF, pneumonia) in the months from December to April (winter to early Spring).

**Conclusion:**

From 2006 to 2016, admission rates of ILD of all causes and IPF subgroup declined but in-hospital mortality of ILD of all causes remained unchanged. Mortality of IPF subgroup and acute respiratory failure subgroup trended down. All-cause hospital admissions and mortality of ILD have a strong seasonal variation. Hospitalization rates for all subgroups (IPF, ARF, pneumonia) were highest in the months from December to April.

## Introduction

Interstitial lung disease (ILD) is a group of lung disorders characterized by abnormalities within the interstitium with or without extensive alteration of alveoli and airways [1]. There have been multiple forms of interstitial lung disease described, most of which lead to progressive lung scarring and dyspnea if left untreated [2, 3]. Idiopathic pulmonary fibrosis (IPF) is one of the most well described ILD with overall very poor prognosis and median survival of 3 to 5 years [4–6]. ILD remains still one of the most challenging respiratory entities to fully understand effectively treat and requires high healthcare utilization. In the past decade, there have been multiple new treatments and knowledge of this complex group of lung disorder. However, study on the overall trend of hospital admission and mortality over the last decade is still needed.

Seasonal variations can play a major role in the general health and wellbeing of patients with respiratory conditions. Winter season can impact lung function and increase the risk of acute exacerbations [7]. The mechanisms of this observation are complex and not fully understood [8]. Pulmonary conditions other than interstitial lung disease such as chronic obstructive pulmonary disease (COPD) have been well studied showing significant seasonal variation [9–11]. Understanding how respiratory diseases change with seasonal variation could guide medical professionals in more effective health resource allocation and to direct future studies on the pathogenesis of this complex entity.

Using a large administrative database, we aimed to analyze the trends and seasonal association of hospital admission and all-cause mortality of ILD in the past 10 years.

## Methods

We obtained the study population from Nationwide Inpatient Sample (NIS) of Agency for Healthcare Resource and Quality (AHRQ) Healthcare Cost and Utilization Project, years 2006 to 2016. All data contained in these database files have previously been de-identified and are off public record, therefore, our institutional review board decided no approval for the study was necessary.

Appropriate weighting was used to produce accurate nation-wide estimates.

Study population was limited to adult patients (age ≥ 18), admitted with the primary diagnosis of interstitial lung disease (ILD) of all causes excluding the identifiable external causes (drug, asbestos, silicosis, pneumoconiosis, hypersensitivity pneumonitis due to organic dusts). (International Classification of Diseases, ninth revision, clinical modification (ICD-9-CM) diagnostic codes 516.30 through 516.37 and 515); ICD-10-CM codes J84.1 through J84.117). A complete list of used ICD codes with description is available in the Appendix. Annual population estimates were obtained from U.S Census Bureau, to account for growing U.S population.

### Statistical analysis

Weighted annual and monthly hospitalization and in-hospital mortality rates were calculated. Hospitalization rates within each year were calculated based on U.S. Census population estimates for a given year. In-hospital mortality rates were calculated with admission number as denominators and in-hospital death numbers as numerators. Monthly hospitalization or mortality rates represent a time series and can be analyzed by seasonal and trend decomposition procedures to reveal long-term trends, seasonality, and random fluctuations. Seasons were defined in a standard manner (“winter” includes December through February, “spring” – March through May, “summer” – June through August, and “fall” – September through November).

We performed multiple subgroup analyses. The first group included all cases with the primary diagnoses of IDL or PF. In second subgroup, we included only record with interstitial pulmonary fibrosis. Interstitial pulmonary fibrosis cases were identified based on the broad case definition algorithm proposed by Raghu et al. (see the Supplementary Appendix 2). In the third subgroup, we included only record of ILD with acute respiratory failure. The fourth subgroup included ILD with concomitant infectious pneumonia. Please se Appendix section for all ICD codes.

Inter-month and inter-seasonal differences of mean number of hospitalizations was assessed by Kruskal-Wallis rank sum test.

Significance and magnitude of observed annual trends was evaluated by Mann-Kendall test and Sen’s slope (Sen PK, 1968).

## Results

### Hospital admissions

**Fig. 1:**
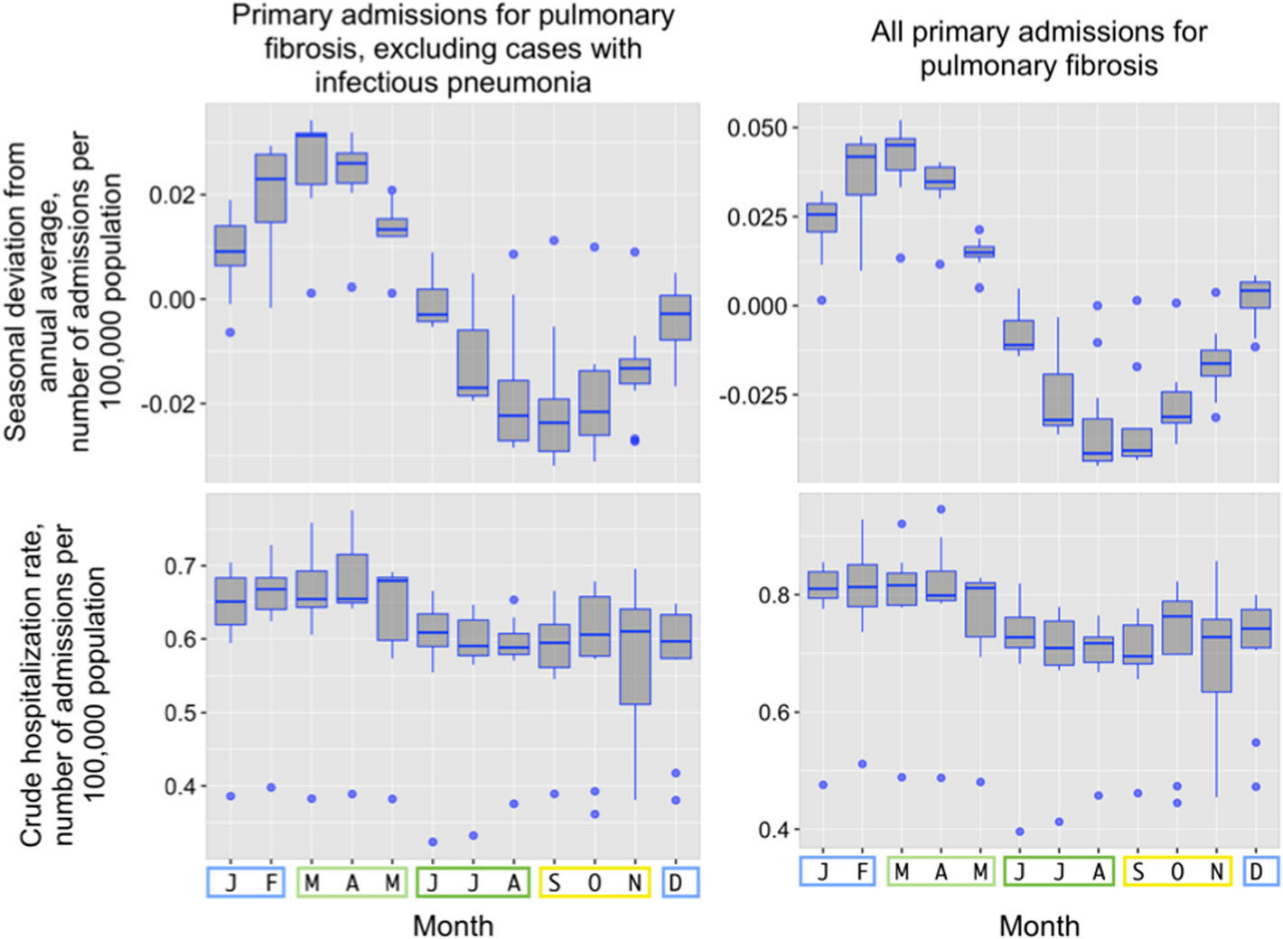
Crude hospitalization rates for interstitial lung disease (primary diagnosis; per 100,000 population) and seasonal deviation from annual average; including and excluding admissions with PNA

Average monthly hospitalization rate per 1,000,000 population ranges from 6.9 ± 0.8 in July to 8 ± 1.2 in April. The months from January to April had higher number of admissions compared to the remaining months of the year (Fig. 1). Seasonal pattern of hospitalization rate was the same between subgroups of included and excluded pneumonia.

**Fig. 2:**
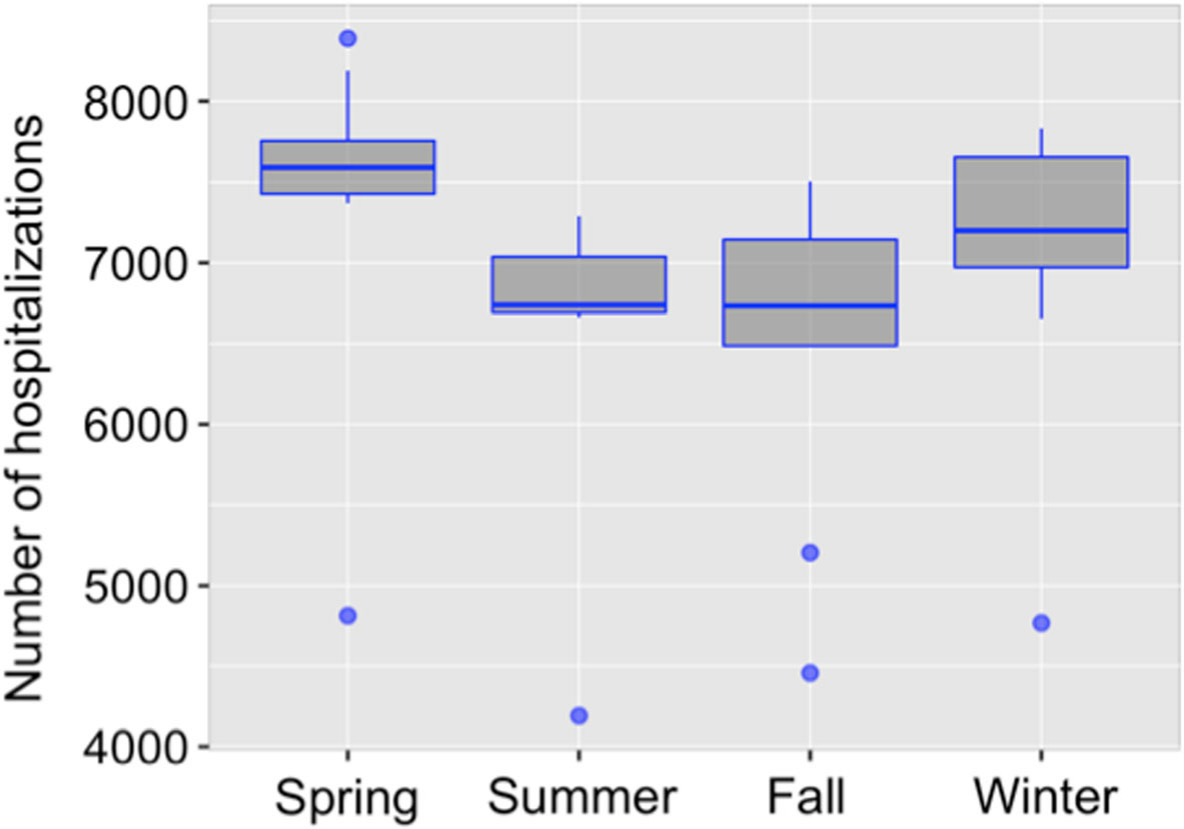
Number of hospitalizations for ILD grouped by season

After merging of months into seasons, mean (±SD) number of hospitalizations in spring, summer, fall and winter were 7447.9 ± 932.0, 6643.0 ± 840.5, 6551.3 ± 922.6 and 7110.3 ± 866.1 respectively (Fig. 2). Inter-seasonal differences did not reach statistical significance (ANOVA *p* = 0.079). However, the difference was found to be significant (independent samples t-test *p*-value = 0.035) by comparison of number of hospitalizations during spring (7447.9 ± 932.0) with other seasons (summer, fall, winter) combined (6768.2 ± 884.8).

**Fig. 3:**
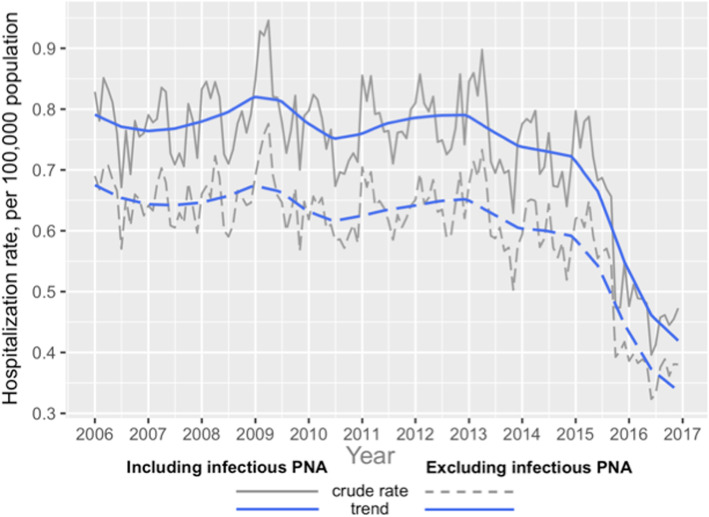
Crude hospitalization rates for ILD and trend (seasonal LOESS decomposition), per 100,000 population, *with* and *without* exclusion of PNA, 2006–2016

Crude monthly hospitalization rate and trend (by LOESS seasonal decomposition) over 11 years (2006–2016) is demonstrated in Fig. 3. The observed descending trends were statistically significant (*p* < 0.001 on Mann-Kendall test) in both subgroups (with and without exclusion of admissions with PNA). Corresponding Sen’s slopes were similar: − 0.00133 in group without exclusion of PNA admissions and − 0.001167 in group with PNA exclusion. Please note, that National Inpatient Sample switched to ICD-10 system in the third quarter of 2015 database, which could affect reporting of multiple diseases and conditions including PF, despite careful translation of ICD-9 diagnostic codes.

### Mortality

The highest mortality was noted in December and February. The presence or absence of diagnosis of infectious pneumonia did not significantly affect seasonal variation of mortality.

**Fig. 4:**
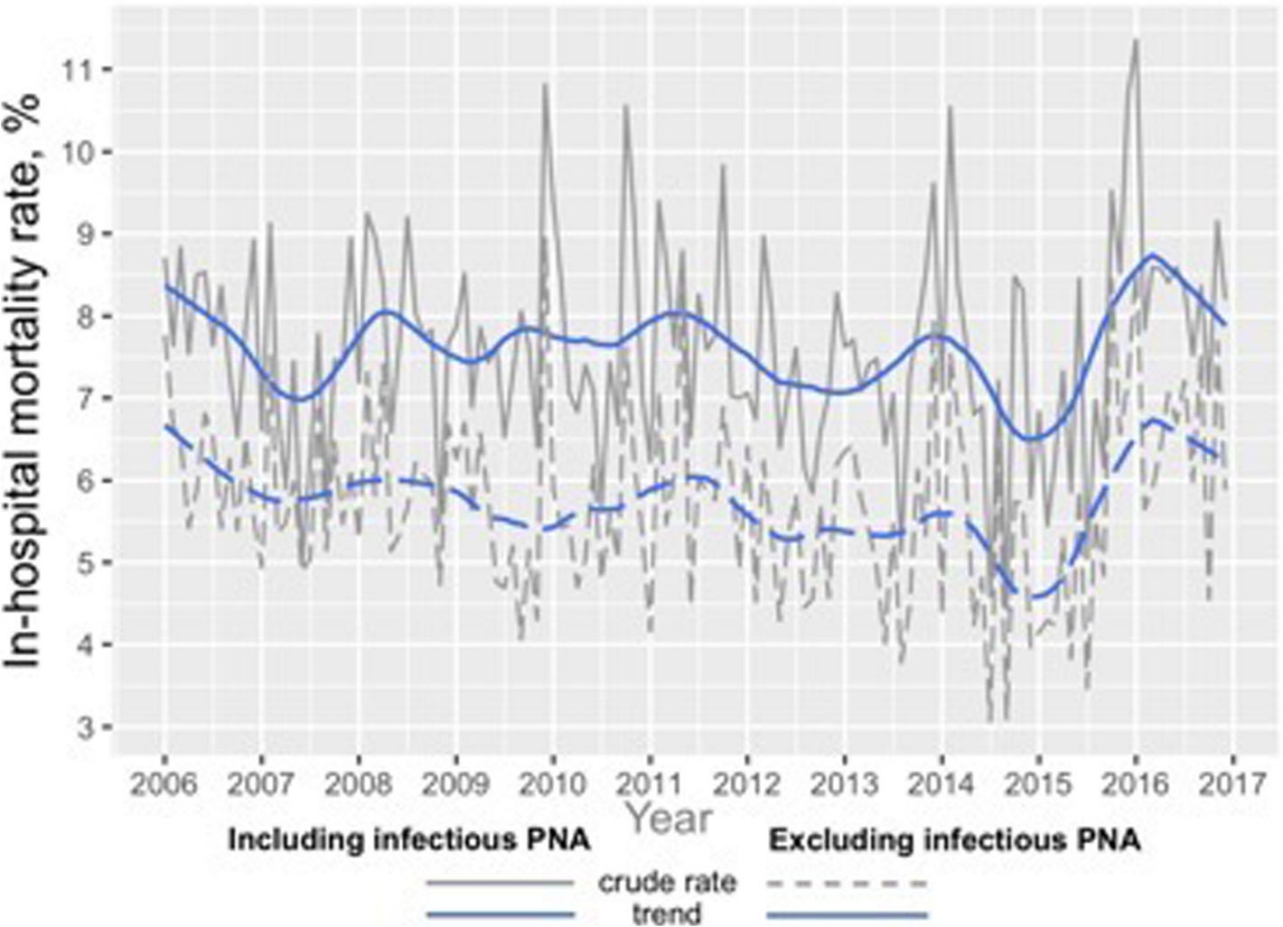
Crude in-hospital mortality rate and trend (seasonal LOESS decomposition), *with* and *without* exclusion of PNA, 2006–2016

Mortality rate in spring, summer, fall and winter were 7.61% ± 0.67, 7.13% ± 0.79, 7.57% ± 0.69 and 8.13% ± 0.60% respectively (Fig. 4). Observed differences were significant (ANOVA *p* = 0.018). Again, the highest mortality predisposition to winter was re-demonstrated.

**Fig. 5:**
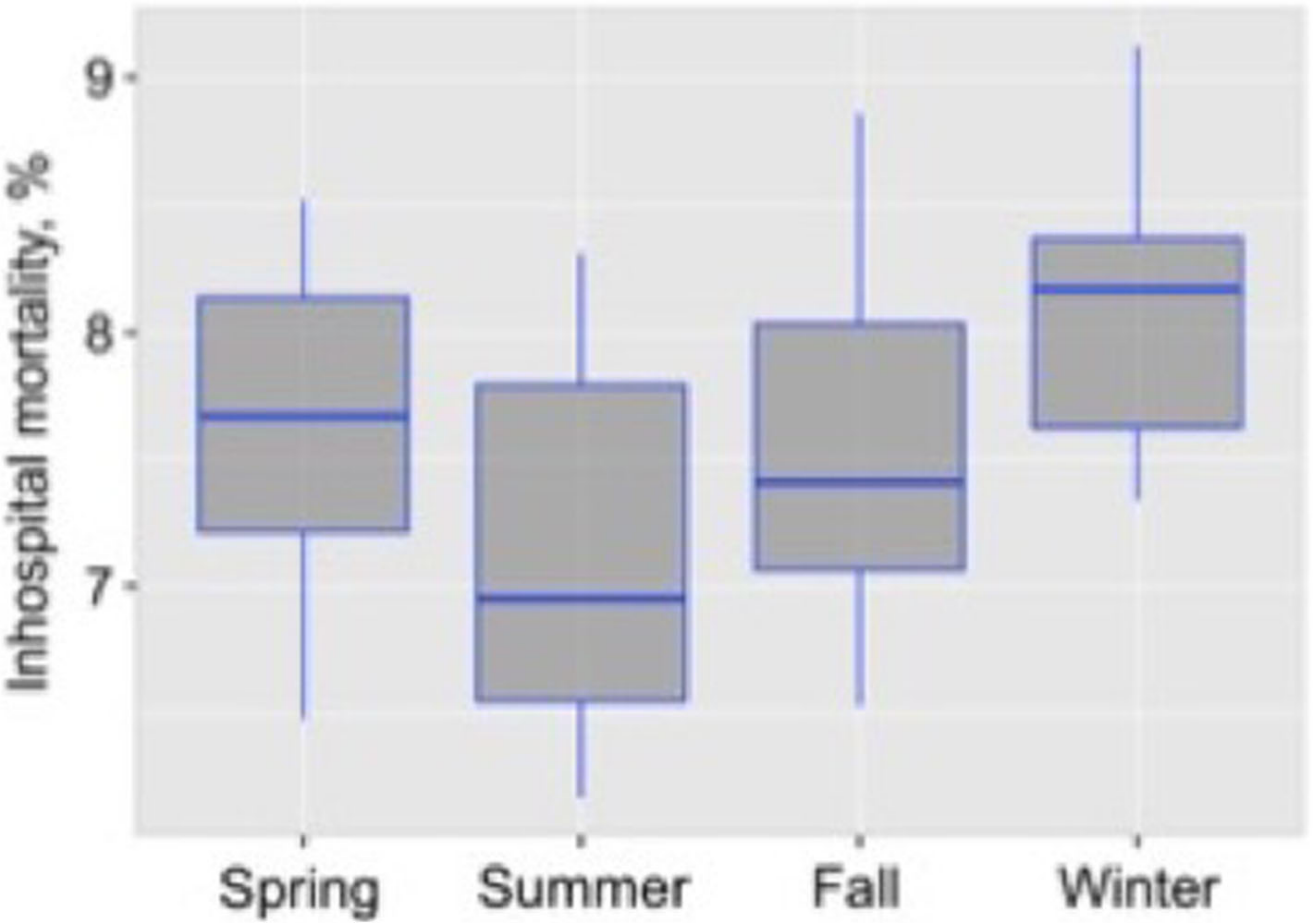
In-hospital mortality by season, %

Trend in mortality rate over 11 years are demonstrated in Fig. 5. Observed trends were not significant (Mann-Kendall *p* = 0.7144 in subgroup without exclusion of PNA admissions, and 0.2218 in subgroup of excluded PNA admissions).

### Subgroups analysis (see figures in supplementary appendix 3)

#### Only idiopathic pulmonary fibrosis (IPF) subgroup

**Fig. 6:**
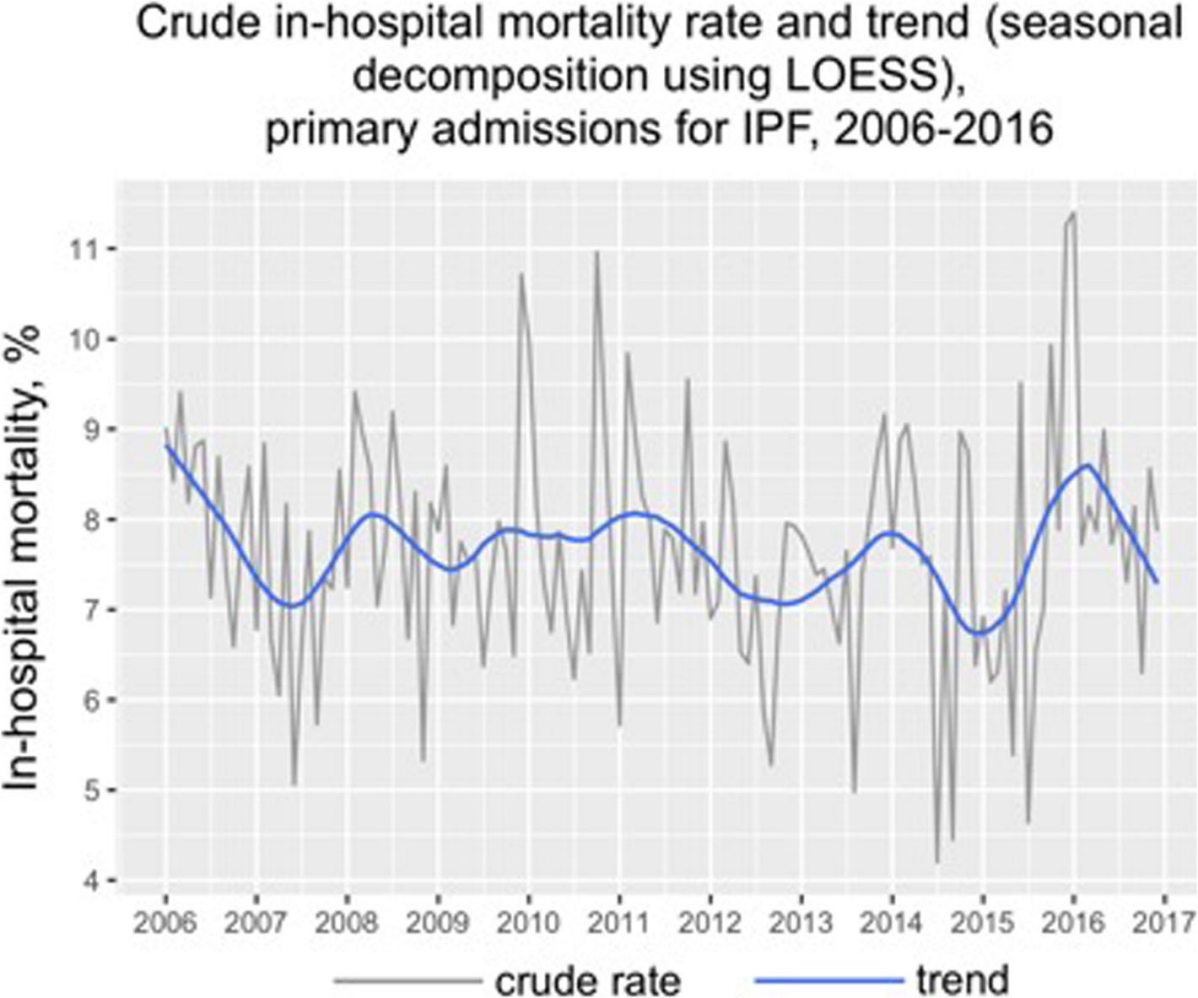
In-hospital mortality rate and trend with seasonal decomposition of primary admissions for IPF, 2006–2016

There was a downward trend in hospitalization and mortality of IPF over 11 years which was statistically significant (*p* < 0.05) (Fig. 6). IPF accounts for 88% of all admission of interstitial lung disease based on our broad diagnosis algorithm. Hospitalization rate of IPF were noted to be highest in the months from January to April compared to the rest of the months but the mortality rates were not different between months.

#### Only acute respiratory failure (ARF) as the cause of admission subgroup

**Fig. 7:**
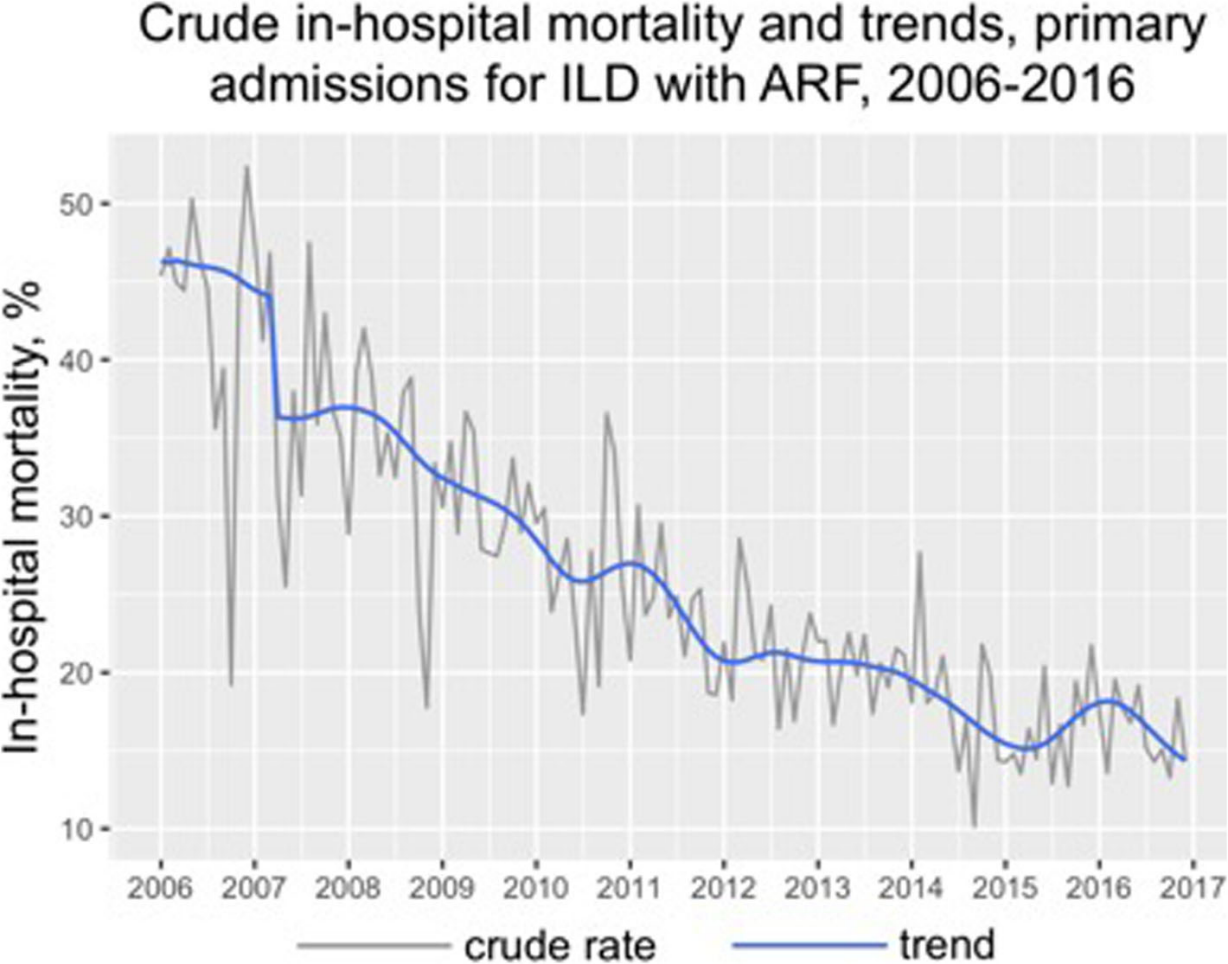
In-hospital mortality rate and trend with seasonal decomposition of primary admissions for ARF, 2006–2016

Acute respiratory failure accounts for 23% of all admission due to interstitial lung disease. Seasonality of hospitalizations and in-hospital mortality did not reach statistical significance on analysis of crude monthly rates, however, seasonal decomposition revealed hidden seasonal variation with slightly higher admission rates in winter. Mortality ranges from 24.2% ± 9.6% in July to 29.1% ± 11% in February. Hospitalization rates demonstrated a strong significant up-going trend with almost three-fold increase during 11 years (trend *p* < 0.001). Mortality decreased at least two-fold with monotonous significant trend (*p* < 0.001) (Fig. 7).

#### **Only pneumonia as the cause of admission subgroup**

Admission with the diagnosis of pneumonia accounts for 17.6% of all admission due to interstitial lung disease. Hospitalization rates in months of December to April were observed to be higher than the remaining months of the year (*p* = 0.007) but mortality did not differ between the months (*p* = 0.876). Hospitalization rate of ILD patients admitted for pneumonia decreased (*p* < 0.05) but mortality rate remained the same in the period from 2006 to 2016 (*p* = 0.756)

## Discussion

To the best of our knowledge, our study is the first to describe both seasonal variations of hospital admission and in-hospital mortality for IPF and non-IPF ILD in the United States in the 11 year- period from 2006 to 2016. Our primary findings are that from 2006 to 2016, all-cause hospital admission rate of patients with interstitial lung disease (all interstitial lung disease and IPF-only subgroup) declined but their overall mortality remained unchanged (except IPF subgroup). Acute respiratory failure related admission account for 23% of all causes and pneumonia 17.6%. Mortality of ILD in general and ILD with acute respiratory failure is highest in winter, up to 8.13% ± 0.60 and 26.3% ± 10.2% respectively. Admission rate for all cause admissions are highest in months from January to April. Subgroup analysis also showed seasonal variations with highest hospitalization rates for all subgroups (IPF, ARF, pneumonia) in the months from December to April (winter to early Spring).

Our finding of highest all-cause mortality for all causes of admissions and subgroup of acute respiratory failure in the winter was similar to the findings by Olson et al. which used a different database for analysis [12]. Seasonal variations were observed in hospitalization rates across all subgroups (acute respiratory failure, IPF, pneumonia) as well. The two most common explanations for winter and early Spring increase in admission rates are respiratory infection and cold temperature. Cold air could hypothetically induce hyperpnoea, subsequently cause drying of the airways [13] and inducing proinflammatory substances production leading to epithelial injury [8]. Infectious etiology was suggested because strong seasonal variations have been reported in COPD, pneumonia and recognized viral illness [14]. There is some evidence that a colder environment could also prolong the life span of viruses. Many viruses such as *influenza A*, RSV and *mycoplasma pneumonia* which cause infections in humans almost exclusively in winter to early spring [15, 16]. One interesting findings is that although winter has highest admission rate for all subgroups (IPF, ARF, pneumonia and ILD in general), the mortality does not have strong seasonal variations in idiopathic pulmonary fibrosis and pneumonia only subgroup. One hypothesis could be the severity of IPF related admissions and pneumonia has no weather association. We could not find literature to explain this finding thoroughly and it could be a topic for future research.

Respiratory causes of death accounted for 64 -89% in patients with ILD [17–19]. We found that acute respiratory failure accounts for 23% of admission of interstitial lung disease and this types of admission has high mortality rate of 26.3% ± 10.2%. This finding concurs with the results of Moua et al. that IPF and non-IPF interstitial lung disease both have very high and similar mortality rates after admission for respiratory distress [20]. Based on a study in Finland, ischemic heart disease, heart failure and lung cancer were the other causes of death [21]. All of those conditions also have been reported to have higher mortality in winter time in the general population [12, 22], which may explain the higher mortality in IPF and non IPF ILD patients in winter time.

Of note, the in-hospital mortality of interstitial lung disease was noted to be significantly higher than the similar study in chronic obstructive lung disease (COPD) and asthma patients using the same national database,8% vs 2, 8% vs 1% respectively [23, 24]. interestingly enough, the mortality rate was 14% higher in the winter compared to the summer, which was less pronounced than the seasonal variations of all cause of deaths of COPD patients (25 to 50% higher in the winter) [12, 25]. Although both COPD and interstitial lung disease are both progressive illnesses with the pathogenesis involving accelerated cellular senescence [26]. This finding suggests that the impact of weather and viral illness on mortality might not be as pronounced in ILD, compared to COPD.

One of the utmost important roles of physicians is to prevent hospital admission for ILD patients. ILD and especially IPF related admissions are significant events after which the lung function of patients will significantly deteriorate with the mean survival only from 2.8 months to 27.7 months [27]. From our study, we found that all cause admission rates in ILD patients, subgroup of only IPF, acute respiratory failure and only pneumonia in the last 11 years were highest in the months December to April (winter to early Spring). Spring in general had highest admission rates compared to the average of other seasons, even when infectious lung diseases were ruled out. Moineddin et al. in their study in the primary care settings found a higher office visits due to respiratory disease in the months from December to April [28].

In the period of 11 years from 2006 to 2016, we observed a decrease in admissions rate for all cause hospital admission for ILD (all types ILD and subgroup of IPF) with the rise in population taken into account. The sharp decrease in 2016 hospital admissions might be a result of incomplete report of administrative data possibly due to the transition from ICD-10 system in the third quarter of 2015. In addition, many advances have been introduced in diagnosis and treatment of interstitial lung disease [29] as well as in hospital management in reducing hospital admissions [30].

The all-cause mortality rate from interstitial lung disease from 2006 to 2016 has been unchanged. However, the all-cause mortality rate of idiopathic pulmonary fibrosis subgroup encouragingly decreased in this 11-year period. Anti-fibrotic treatment availability could be a possible explanation. A recent large database study by Demsey et al. reported a decreased mortality risk in IPF patients in the first 2 years of anti-fibrotic treatment [31]. However, it is challenging to pinpoint a single factor that lead to this encouraging result based on our study especially when antifibrotic therapies were only approved since 2014 [32].

Our study has limitations. We did not include all types of interstitial lung disease We excluded the interstitial lung disease group with identifiable external agents (organic dust, drug, asbestos, silicosis, pneumoconiosis) because of two reasons. Firstly, it is for the comparison with the results of the study by Olson et al. for the interstitial lung disease group from 1992 to 2003 [12] and secondly, including ILD group with identifiable external agents with different pathogenesis will create more heterogeneity to our population. IPF cases were identified based on a broad definition algorithm which has been commonly used in the epidemiology studies. However, this algorithm was sensitive but not specific [33], thus could overestimate the prevalence of IPF in our ILD population. We also did not include the analysis of ILD with and without lung cancer subgroups because it would require extensive analysis beyond the scope of this manuscript. Concomitant lung cancer and interstitial lung disease could be a topic for future studies. Although we have included all ICD-9-CM and ICD-10-CM codes for interstitial lung disease, the results are inevitably susceptible to errors from coding inaccuracies. Nevertheless, this study has provided with an important and objective overview on the seasonal variations and trends in admissions and mortality of this entity spectrum over a long period of time.

## Conclusion

All cause hospital admission and mortality of interstitial lung disease have a strong seasonal variation in 11 years from 2006 to 2016. Hospital admissions are highest in the period from January to May, in-hospital death was highest in the winter. All- cause hospital admission of patients with interstitial lung disease declined but their mortality remained unchanged, with or without the presence of infectious pneumonia.

## Supplementary information

**Additional file 1.**

**Additional file 2.**

## Data Availability

All data were stored in the national inpatient sample database.

## Abbreviation

ILD: Interstitial Lung Disease
IPF: Idiopathic Pulmonary Fibrosis
PF: Pulmonary Fibrosis
NIS: Nationwide Inpatient Sample
ICD-9CM: International Classification of Diseases, ninth revision, clinical modification
