# Supplementary material for: Trends and seasonal variation of hospitalization and mortality of interstitial lung disease in the United States from 2006 to 2016
Source: Respir Res. 2020 Jun 16;21:152. doi: 10.1186/s12931-020-01421-0 (PMC7298940; doi:10.1186/s12931-020-01421-0)
Supplement: Supplementary file 1 — Additional file 1. [file 12931_2020_1421_MOESM1_ESM.docx]

SUPPLEMENT APPENDIX 2

1/ Idiopathic interstitial fibrosis algorithm

ICD 9: 516.3 + 516.31 +515

WITHOUT concomitant codes

135 Sarcoidosis

237·7 Neurofibromatosis

272·7 Lipidoses

277·3 Amyloidosis

277·8 Other specified disorders of metabolism – includes eosinophilic granuloma

446·21 Goodpasture’s syndrome

446·4 Wegener’s granulomatosis

495 Extrinsic allergic alveolitis

500 Coal workers’ pneumoconiosis

501 Asbestosis

502 Pneumoconiosis due to other silica or silicates

503 Pneumoconiosis due to other inorganic dust

504 Pneumoconiosis due to inhalation of other dust

505 Pneumoconiosis, unspecified

506·4 Chronic respiratory conditions due to fumes or vapors

508·1 Chronic and other pulmonary manifestations due to radiation

508·8 Respiratory conditions due to other specified external agents

516·0 Pulmonary alveolar proteinosis

516·1 Idiopathic pulmonary hemosiderosis

516·2 Pulmonary alveolar microlithiasis

516·8 Other specified alveolar and parietoalveolar pneumonopathies

516·9 Unspecified alveolar and parietoalveolar pneumonopathies

517·0 Lung involvement in conditions classified elsewhere

517·2 Lung involvement in systemic sclerosis

517·8 Lung involvement in other diseases classified elsewhere

518·3 Pulmonary eosinophilia

555 Regional enteritis

710·0 Systemic lupus erythematosus

710·1 Systemic sclerosis

710·2 Sjögren’s disease

710·3 Dermatomyositis

710·4 Polymyositis

714·81 Rheumatoid lung

720 Ankylosing spondylitis

759·5 Tuberous sclerosis

ICD 10:

J84.1, J84.11, J84.112, J84.10

WITHOUT concomitant codes

D86.9

Q85.00

E75.6

E85.9

E88.89

M31.0

M31.3

J67.9

J61

J60

J62

J64

J63

J68.4

J70.1

J70.8

J84.01

J84.02

J84.03

J84.09

J82

K50

M32

M34

M35.0

M33

M33.2

M05.10

M45.9

Q85.1

2/ Acute respiratory failure codes

**acute respiratory failure (ICD 10: J96.00, J96.02,J96.0, J96.01, J96.2,J96.20,J96.21, J96.22, J96.2 ;  ICD 9: 518.51, 518.81, 518.53, 518.84),**
