# Supplementary material for: Trends and seasonal variation of hospitalization and mortality of interstitial lung disease in the United States from 2006 to 2016
Source: Respir Res. 2020 Jun 16;21:152. doi: 10.1186/s12931-020-01421-0 (PMC7298940; doi:10.1186/s12931-020-01421-0)

| **Months** | **Number of hospitalizations, monthly**  **(mean ± SD)** | **Monthly hospitalization rate, per 100,000 population**  **(mean ± SD)** | **In-hospital mortality, % (mean ± SD)** |
| --- | --- | --- | --- |
| **Jan** | 2,437 ± 312 | 0.79 ± 0.11 | 7.81 ± 1.49 |
| **Feb** | 2,456 ± 313 | 0.79 ± 0.11 | 8.23 ± 1.39 |
| **Mar** | 2,458 ± 315 | 0.79 ± 0.11 | 7.87 ± 1.04 |
| **Apr** | 2,482 ± 337 | 0.80 ± 0.12 | 7.56 ± 0.73 |
| **May** | 2,353 ± 293 | 0.76 ± 0.10 | 7.37 ± 0.93 |
| **Jun** | 2,202 ± 321 | 0.71 ± 0.11 | 7.25 ± 1.09 |
| **Jul** | 2,151 ± 304 | 0.69 ± 0.10 | 6.92 ± 1.45 |
| **Aug** | 2,149 ± 227 | 0.69 ± 0.08 | 7.23 ± 0.90 |
| **Sep** | 2,154 ± 245 | 0.69 ± 0.09 | 6.85 ± 1.20 |
| **Oct** | 2,197 ± 368 | 0.71 ± 0.13 | 8.12 ± 1.33 |
| **Nov** | 2,131 ± 363 | 0.68 ± 0.13 | 7.72 ± 1.17 |
| **Dec** | 2,207 ± 296 | 0.71 ± 0.10 | 8.45 ± 1.56 |

1/ Table 1: ILD hospitalization rates and in-hospital mortality by month

2/ Subgroup analysis of only idiopathic pulmonary fibrosis


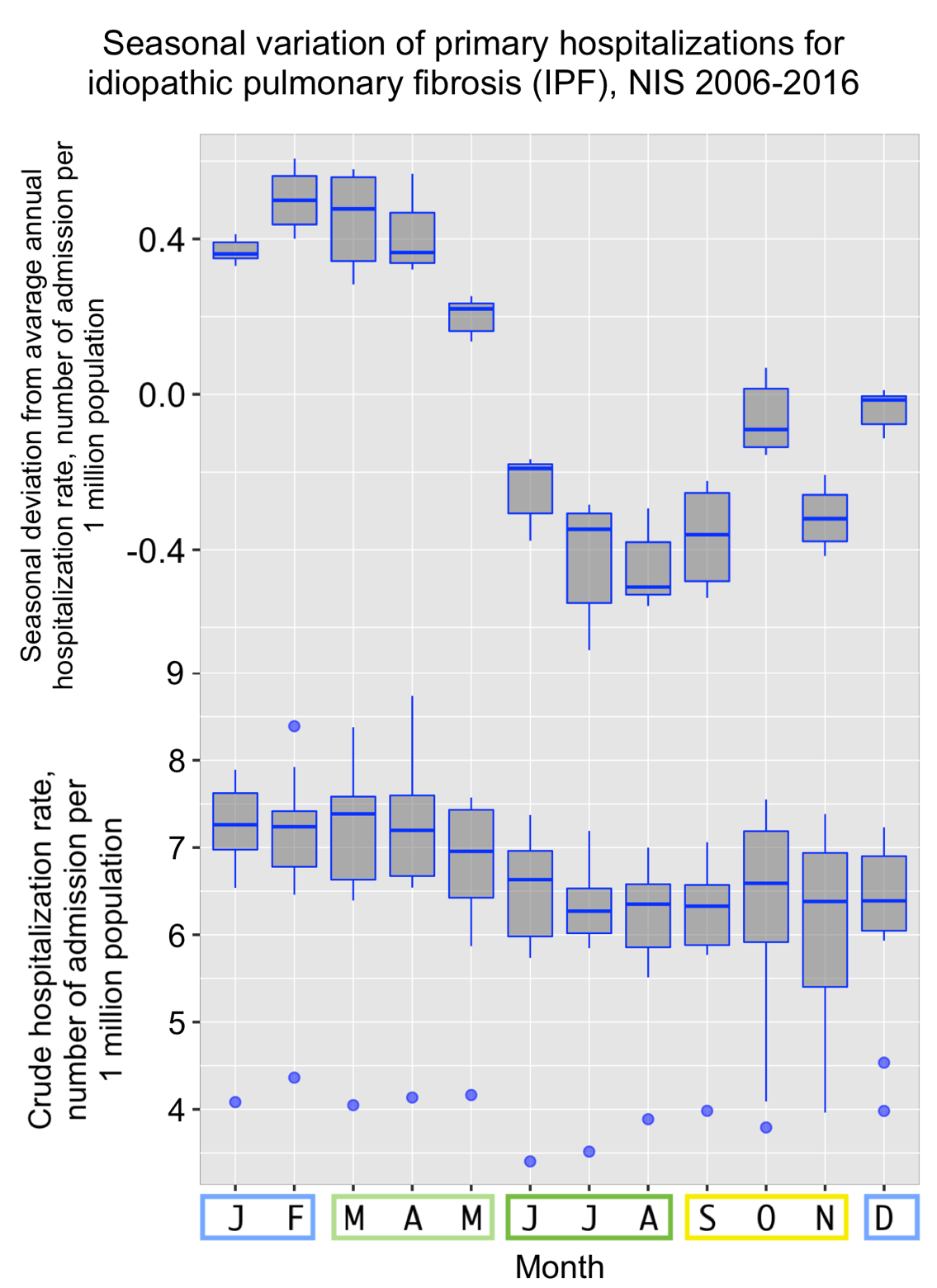


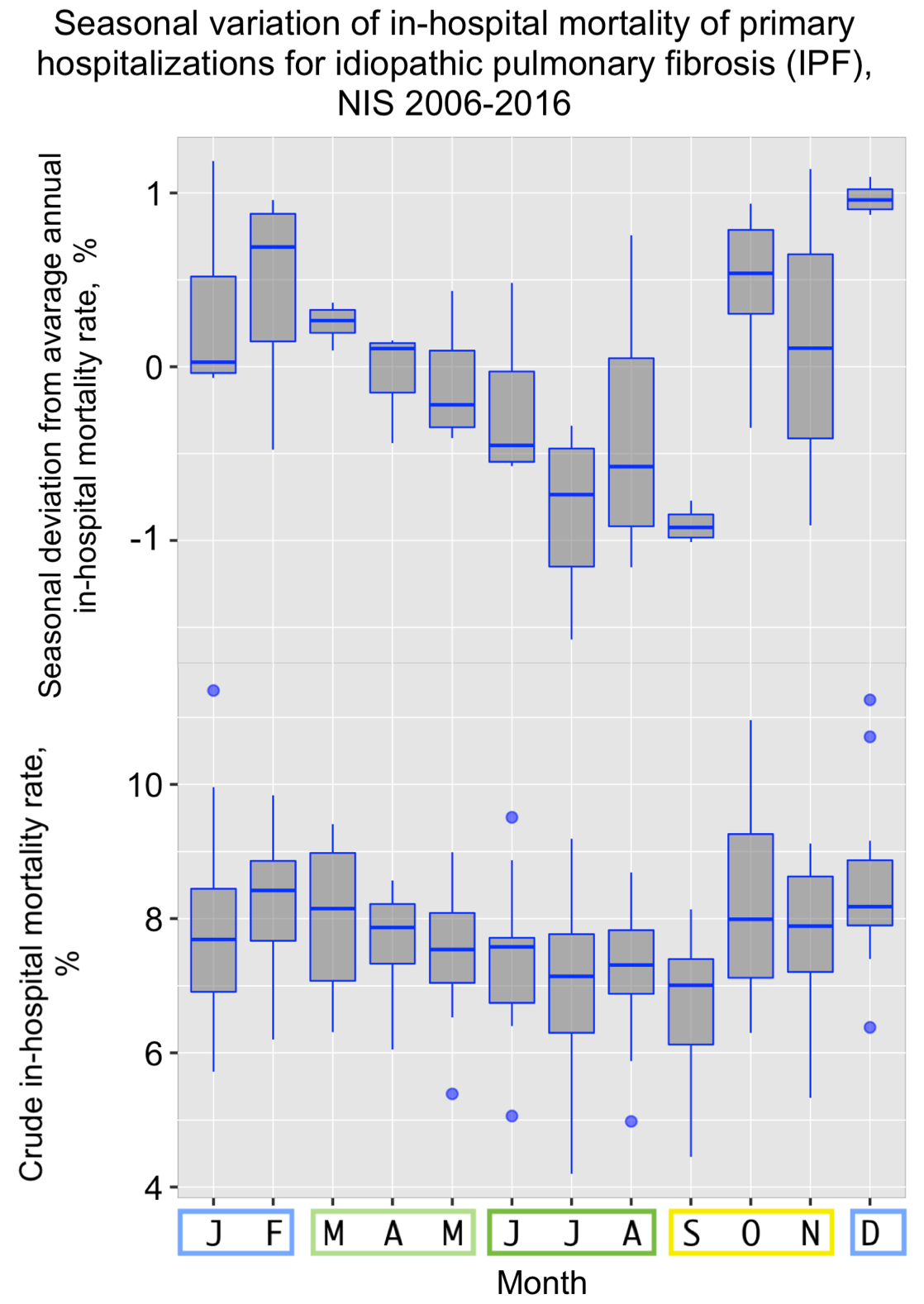


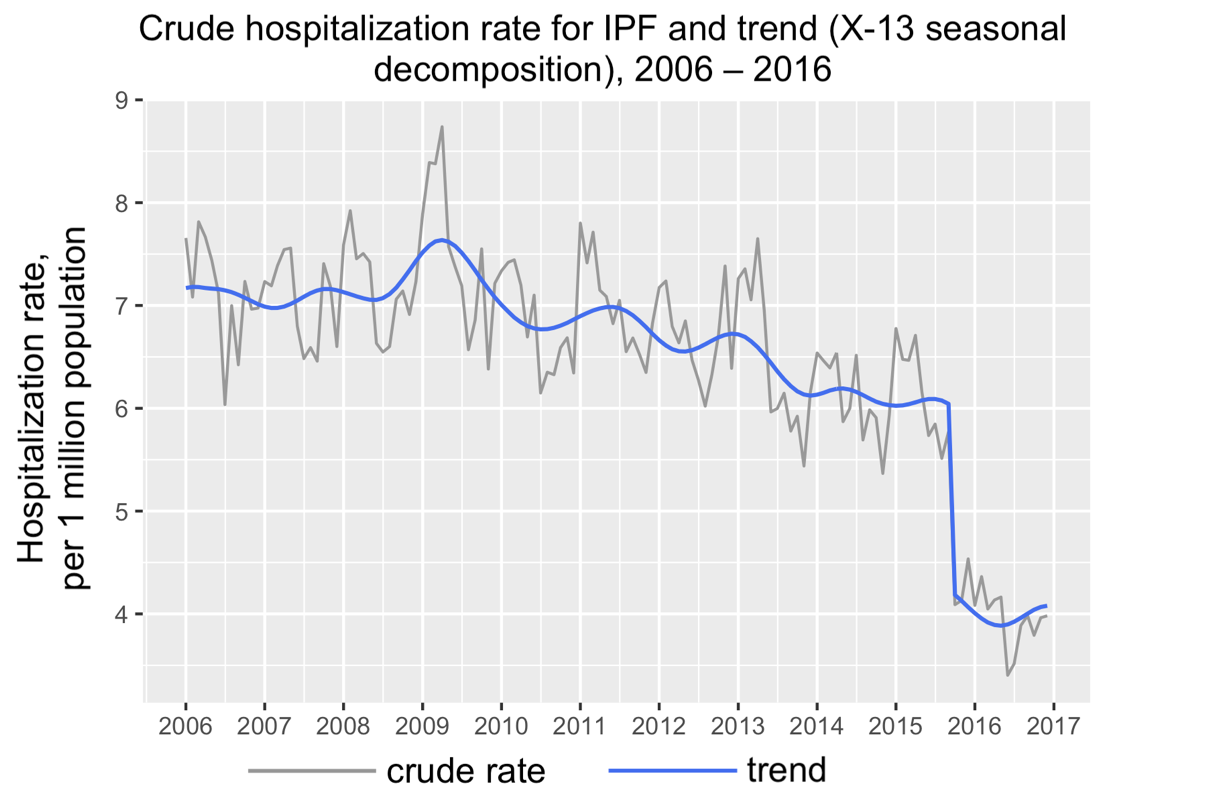


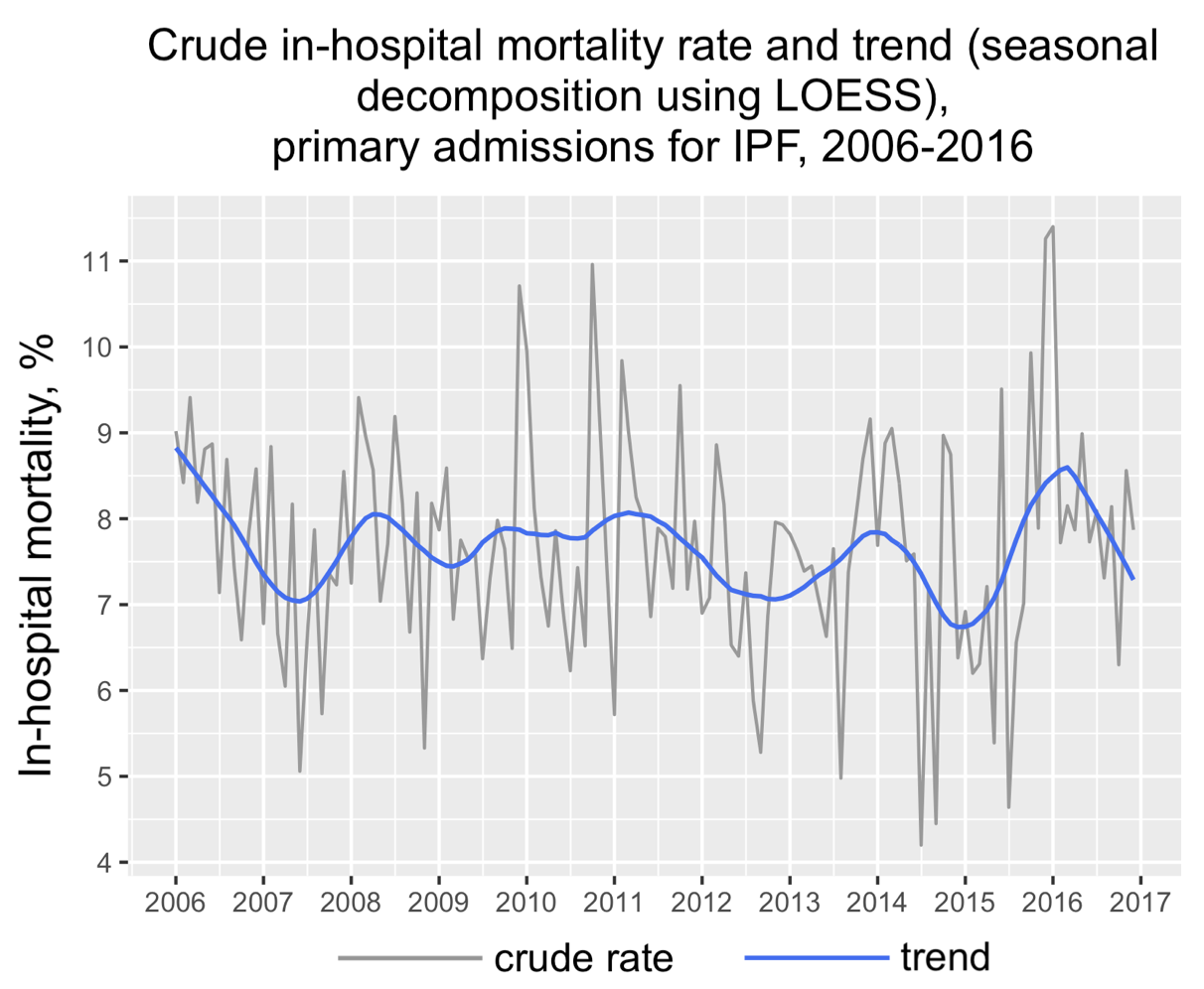


2/ Subgroup analysis of only pneumonia


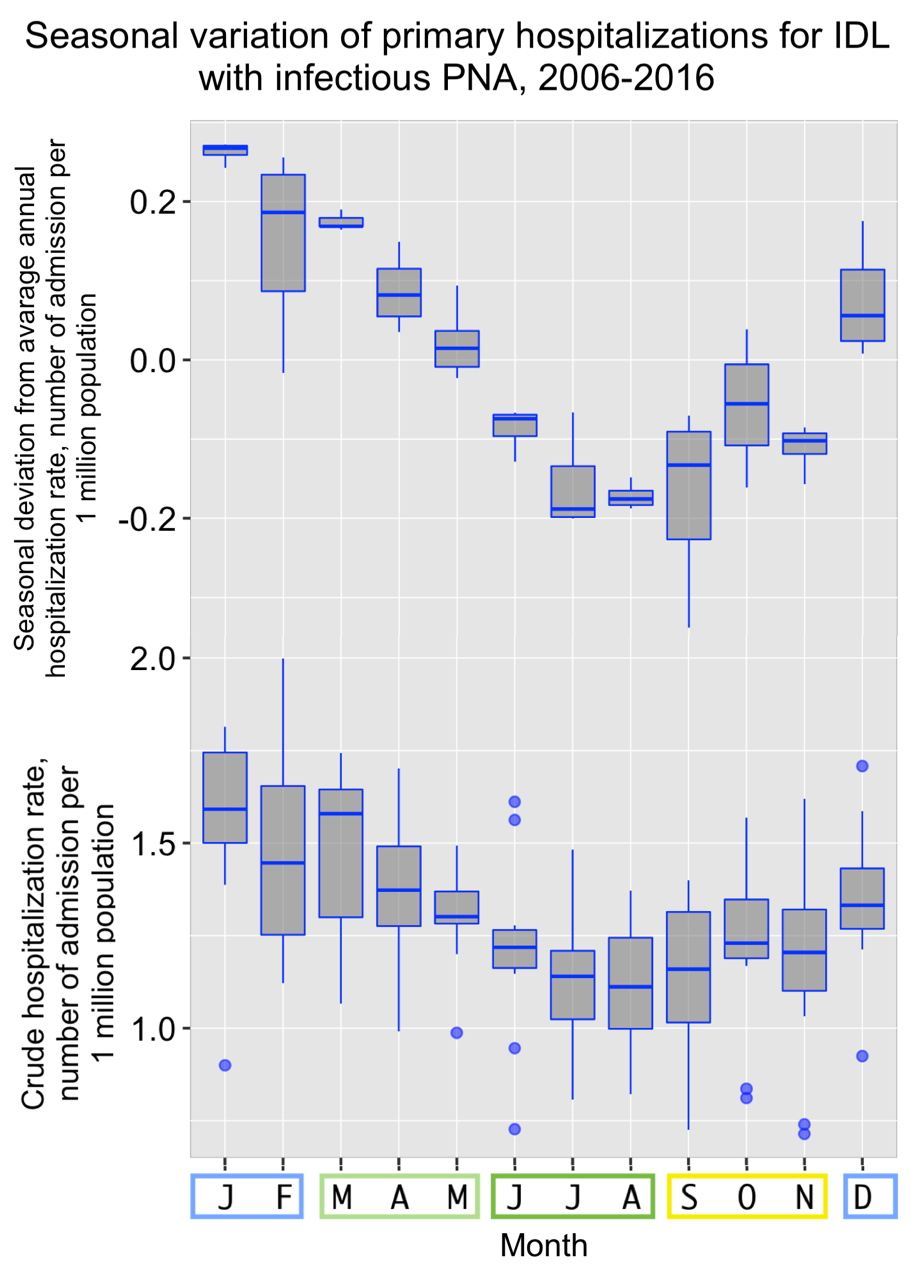


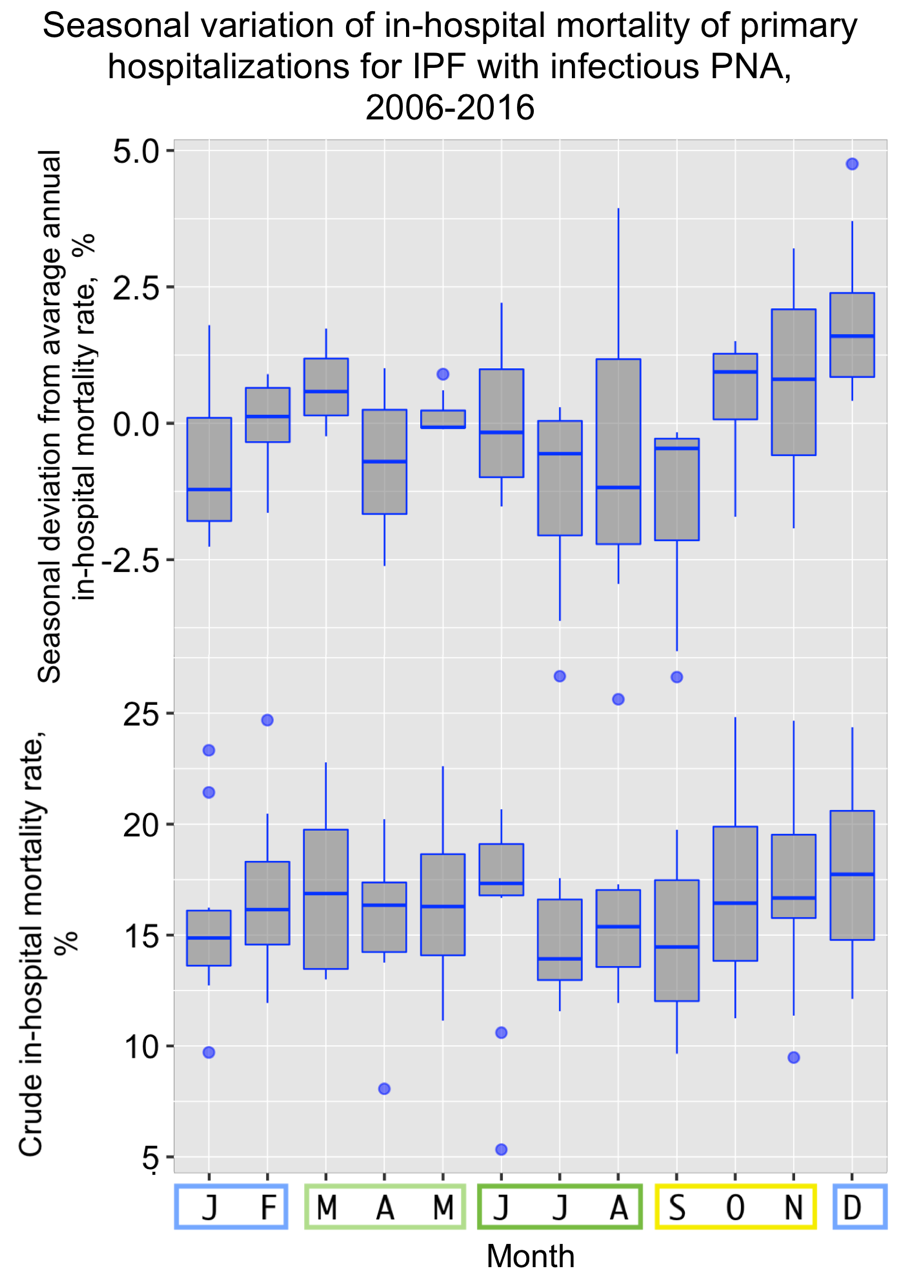


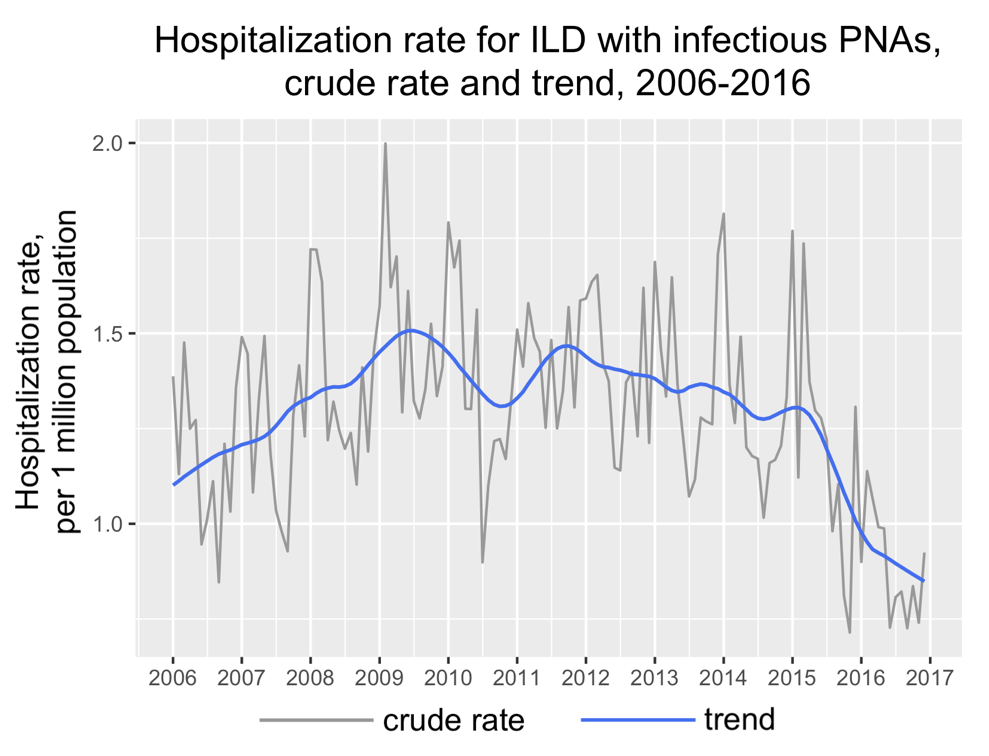


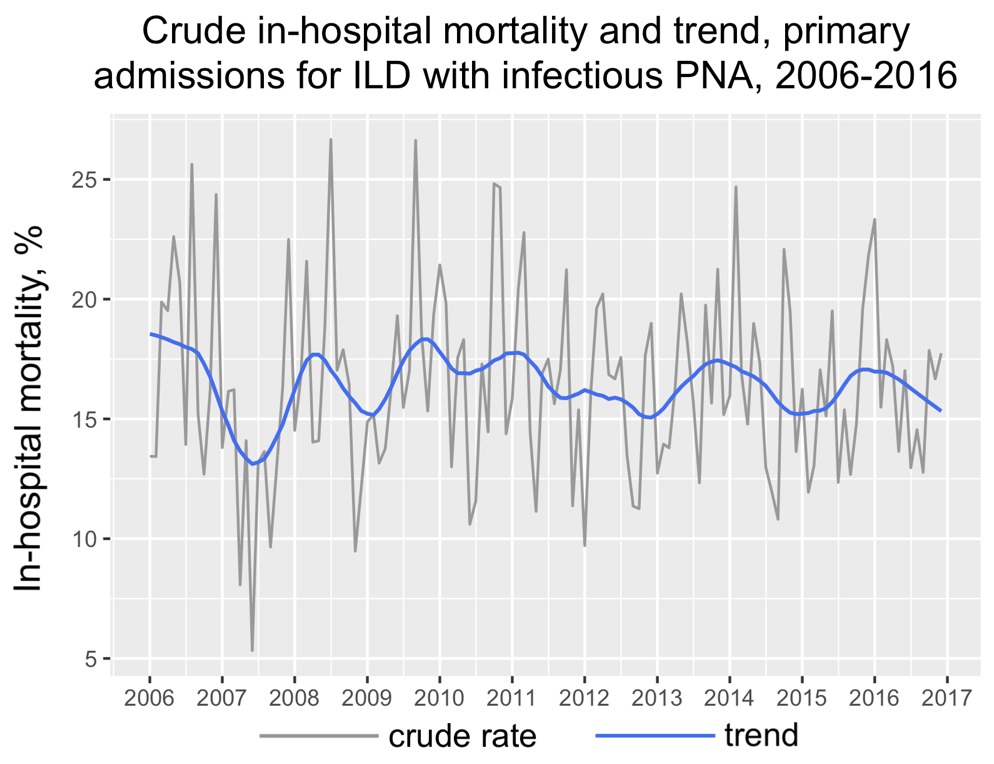


3/ Subgroup analysis of admissions due to acute respiratory failure


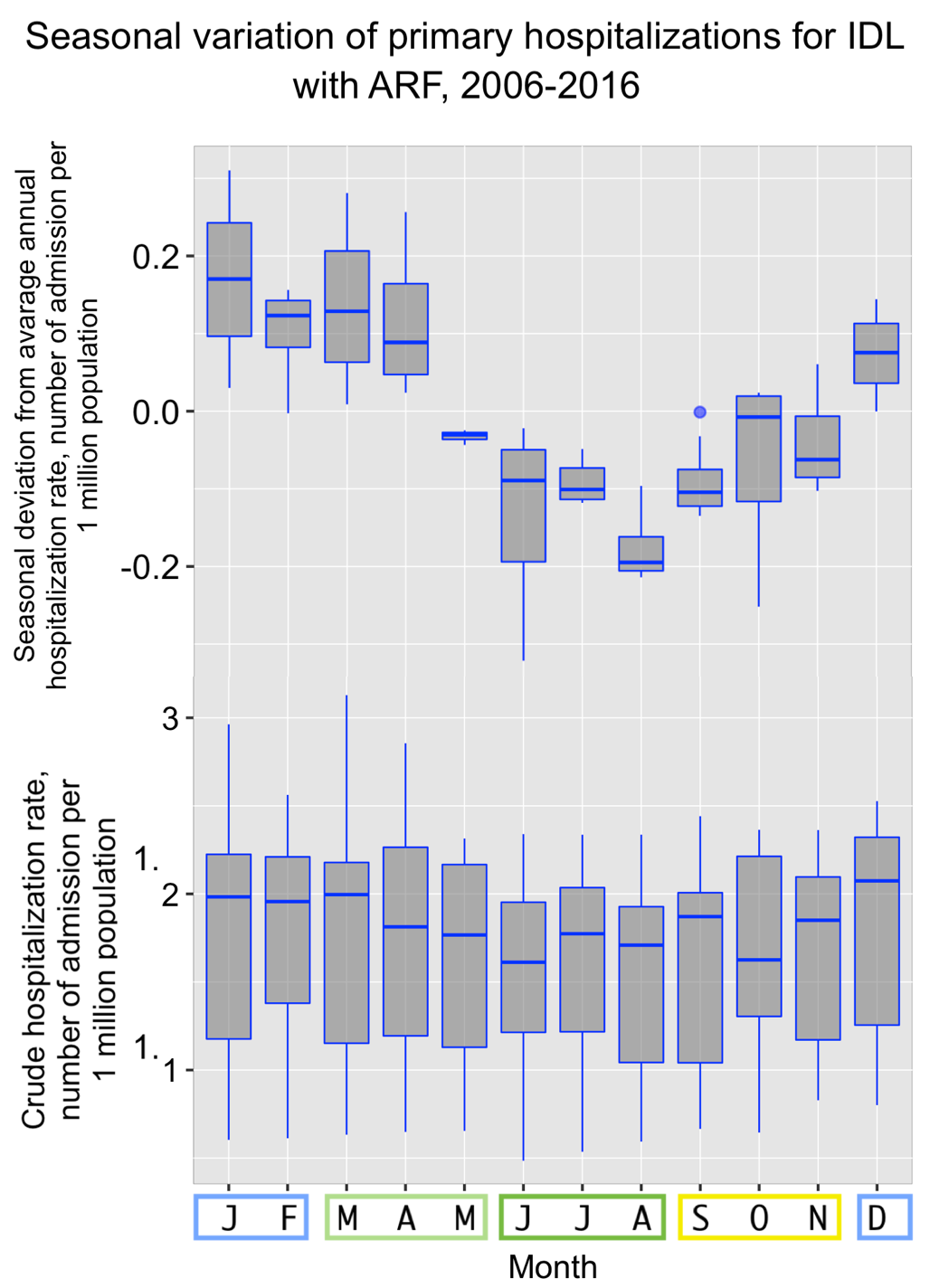


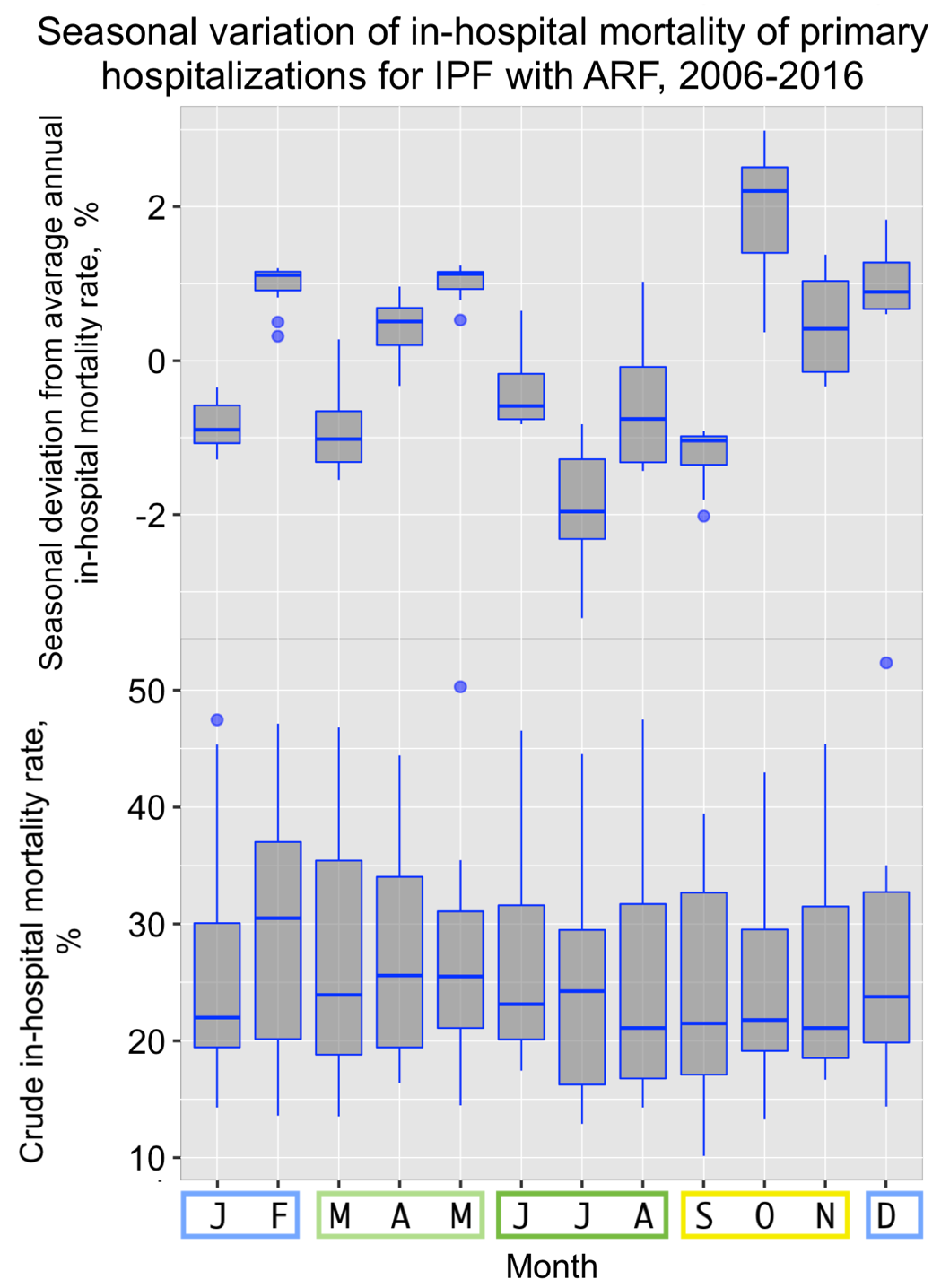


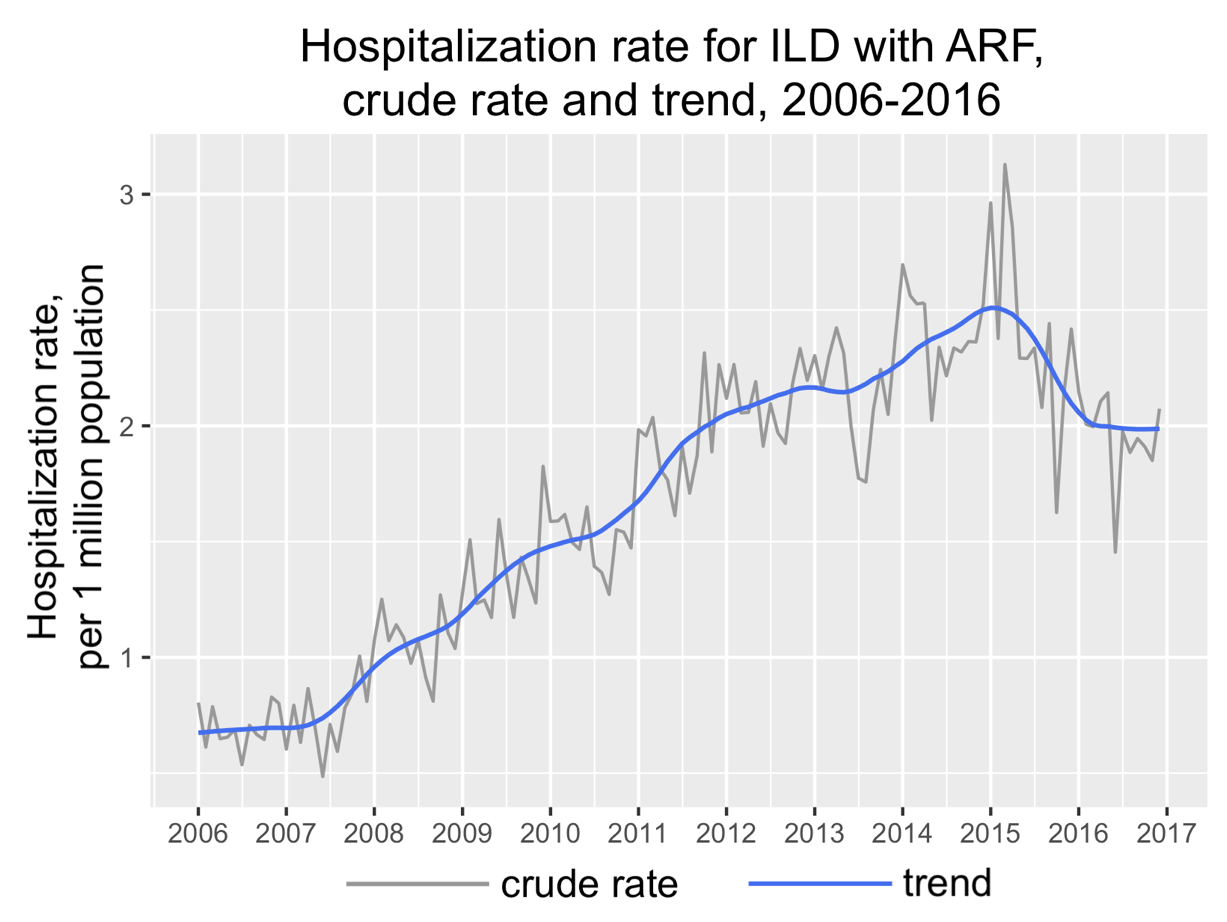


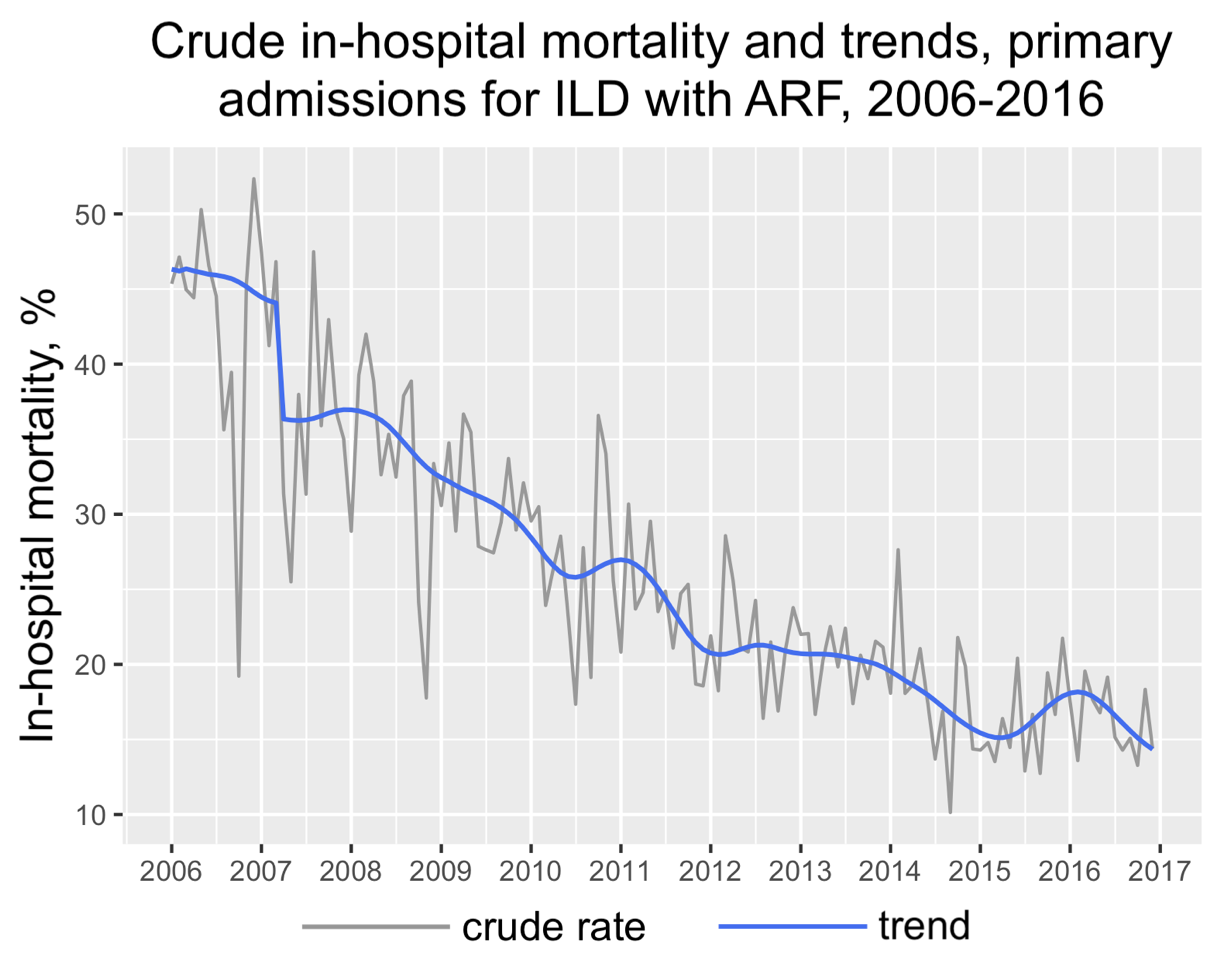

Supplement: Supplementary file 2 — Additional file 2. [file 12931_2020_1421_MOESM2_ESM.docx]
